# Sinonasal organising haematoma – a little known entity^☆^

**DOI:** 10.1016/j.bjorl.2018.05.013

**Published:** 2018-07-17

**Authors:** Lalee Varghese, Sramana Mukhopadhyay, Raghav Mehan, Regi Kurien, Meera Thomas, V. Rupa

**Affiliations:** a Christian Medical College, Department of Otorhinolaryngology, Vellore, India; b Christian Medical College, Department of Pathology, Vellore, India

**Keywords:** Haematoma, Paranasal sinuses, Unilateral, Neoplasms, Benign, Epistaxis, Surgery, Haematoma em organização, Seios paranasais, Massa nasal unilateral

## Abstract

**Introduction:**

Sinonasal organising haematoma is a recently described, rare, benign inflammatory condition, which closely resembles malignancy in its clinical presentation.

**Objective:**

To describe the clinical features of organising haematoma and to review the evolution of surgical options successfully used.

**Methods:**

A retrospective review of charts of all patients with a histopathological diagnosis of sinonasal organising haematoma was performed.

**Results:**

Six (60%) of the 10 patients were male with a mean age of 47.4 years. All patients had unilateral disease with recurrent epistaxis as the presenting symptom. Maxillary sinus was the most commonly involved sinus. There was no history of trauma in any of the patients. Hypertension (80%) was the most commonly associated comorbidity. Contrast-enhanced CT scan of the paranasal sinuses showed heterogeneous sinus opacification with/without bone erosion. Histopathological examination was diagnostic. Complete endoscopic excision was done in all patients resulting in resolution of the disease.

**Conclusion:**

Awareness of this relatively new clinical entity and its evaluation and treatment is important for otolaryngologists, maxillofacial surgeons and pathologists alike. Despite the clinical picture of malignancy, histopathological features of benign disease can safely dispel such a diagnosis.

## Resumo

**Introdução:**

Haematoma nasossinusal em organização é uma condição inflamatória benigna rara, recentemente descrita, que se assemelha a lesões malignas em sua apresentação clínica.

**Objetivo:**

Descrever as características clínicas do haematoma em organização e analisar a evolução das opções cirúrgicas utilizadas com sucesso.

**Método:**

Foi realizada a revisão retrospectiva dos prontuários de todos os pacientes com diagnóstico histopatológico de haematoma nasossinusal em organização.

**Resultados:**

Seis (60%) dos 10 pacientes eram do sexo masculino, com média de idade de 47,4 anos. Todos os pacientes apresentavam doença unilateral com epistaxe recorrente como sintoma de apresentação. O seio maxilar era o mais comumente afetado. Não havia histórico de trauma em nenhum dos pacientes. Hipertensão (80%) foi a comorbidade mais comumente associada. A tomografia computadorizada dos seios paranasais com contraste mostrou opacificação heterogênea do seio com/sem erosão óssea. O exame histopatológico foi diagnóstico. A excisão endoscópica completa foi realizada em todos os pacientes, resultando na resolução da doença.

**Conclusão:**

A conscientização a respeito desta entidade clínica relativamente nova e sua avaliação e tratamento é importante para os otorrinolaringologistas, cirurgiões buco-maxilo-faciais e patologistas. Apesar do quadro clínico de malignidade, as características histopatológicas da doença benigna podem descartar com segurança esse diagnóstico.

## Introduction

Sinonasal organising haematoma (OH) is an uncommon, non-neoplastic condition, which is locally aggressive. It was first reported in Japanese literature in 1917 by Tadokoro as a “blood boil of the maxillary sinus”.^1^ It has also been referred to as haematoma,^2^ haematoma-like mass,^1^ pseudotumour^3^ or organising haematoma of the maxillary sinus.^4^ The maxillary sinus is known to be the most commonly involved sinus.^5^, ^6^ The aetiopathogenesis of this entity is still ambiguous.

The disease is mostly unilateral and usually presents with nasal obstruction and epistaxis.^4^, ^5^ Contrast-enhanced CT scan of the paranasal sinuses may reveal bony destruction, erosions and heterogeneous soft tissue densities in the involved sinuses. The close resemblance of these radiological findings to a maxillary sinus malignancy creates a diagnostic dilemma. Multiple biopsies are often performed because they often result in a “negative biopsy”. Complete surgical excision either by an endoscopic or a combined sublabial and endoscopic approach is the definitive treatment for OH. Total excision also enables the pathologist to systematically exclude other pathological diagnoses which may present in a similar manner.

Awareness regarding OH is still very low, both among the clinicians as well as the pathologists. In the present report, we aimed to study the clinical profile, management and treatment outcomes of all patients diagnosed with sinonasal OH seen over the last 6 years.

## Methods

We conducted a retrospective chart review of patients who were diagnosed with OH between 2010 and 2016 at a tertiary care hospital in South India. Data regarding demography, clinical features, radiology, histopathology, treatment and follow up was collected and analysed. The study was approved by the Institutional Review Board.

## Results

### Demography

A total of 10 patients were diagnosed with OH during the study period. Most (60%) patients were males. The mean age at presentation was 47.4 ± 12.3 years (range 28–63 years). All patients had unilateral disease. There was no side predilection, pathology being present on the right side in five patients and on the left side in another five patients. The duration of symptoms ranged from 2 months to 12 years (mean = 28.4 months) (Table 1).

**Table 1:** Demographics and clinical profile of the study patients (*n* = 10).

Case no.	Age (years)/Sex	Side	Symptoms	Duration of symptoms (months)	Nasal endoscopy findings	Comorbidities
1	54/M	R	Nasal obstruction, epistaxis	12	Bulging of lateral wall, Mass in MM	HTN, Factor XI deficiency
2	32/M	R	Nasal obstruction, epistaxis, headache, periorbital swelling, epiphora	144	Mass filling nasal cavity	Nil
3	57/F	R	Nasal obstruction, epistaxis, facial pain	6	Bulging of lateral wall	HTN
4	28/M	L	Nasal obstruction, epistaxis	48	Blood stained discharge in MM	Nil
5	63/M	L	Nasal obstruction, blood stained discharge cheek swelling	12	Bulging of lateral wall	HTN
6	52/M	L	Epistaxis	2	Blood clot in MM	HTN Thrombocytopenia
7	55/M	L	Nasal obstruction, epistaxis, nasal mass	24	Black fleshy friable mass	HTN
8	57/F	R	Epistaxis	18	Fleshy mass	HTN, DM
9	42/F	R	Nasal obstruction, epistaxis, headache, cheek numbness	6	Fleshy polypoidal gritty mass	HTN, DM
10	34/F	L	Nasal obstruction, epistaxis, headache	12	Fleshy vascular mass	HTN

M, Male; F, Female; R, Right; L, Left; MM, Middle Meatus; HTN, Hypertension, DM, Diabetes mellitus.

### Clinical features

Most (80%) patients had ipsilateral nasal obstruction. There was a history of recurrent epistaxis in all patients, with only one patient having blood-stained nasal discharge and the rest having moderate epistaxis. One patient complained of periorbital swelling and epiphora of recent onset. Headache, facial pain, cheek swelling and numbness were the other symptoms that patients complained of.

Hypertension was the most common (80%) comorbidity. One patient suffered from mild factor XI deficiency which was indicated by a deranged activated partial thromboplastin time (APTT). This patient also had hypertension. One patient had associated idiopathic thrombocytopenia, which was corrected before the surgical intervention. None of the patients were taking anticoagulant drugs. Three patients had undergone endoscopic sinus surgery at another centre before presentation, but two patients had no biopsy report. In the third patient the report was of a benign polyp.

Preoperative rigid nasal endoscopy revealed a vascular nasal mass in six patients (60%) and bulging of the lateral nasal wall in three patients (30%). Two patients did not have any of the above features, but showed blood stained discharge or blood clot in the middle meatus. Biopsy was done before the definitive surgical excision in seven (70%) patients, none of which was suggestive of malignancy. In four patients pre-operative histopathological examination was suggestive of OH whereas in the other three it was reported as fibrinous exudate and no viable tissue.

### Radiology

**Figure 1:**
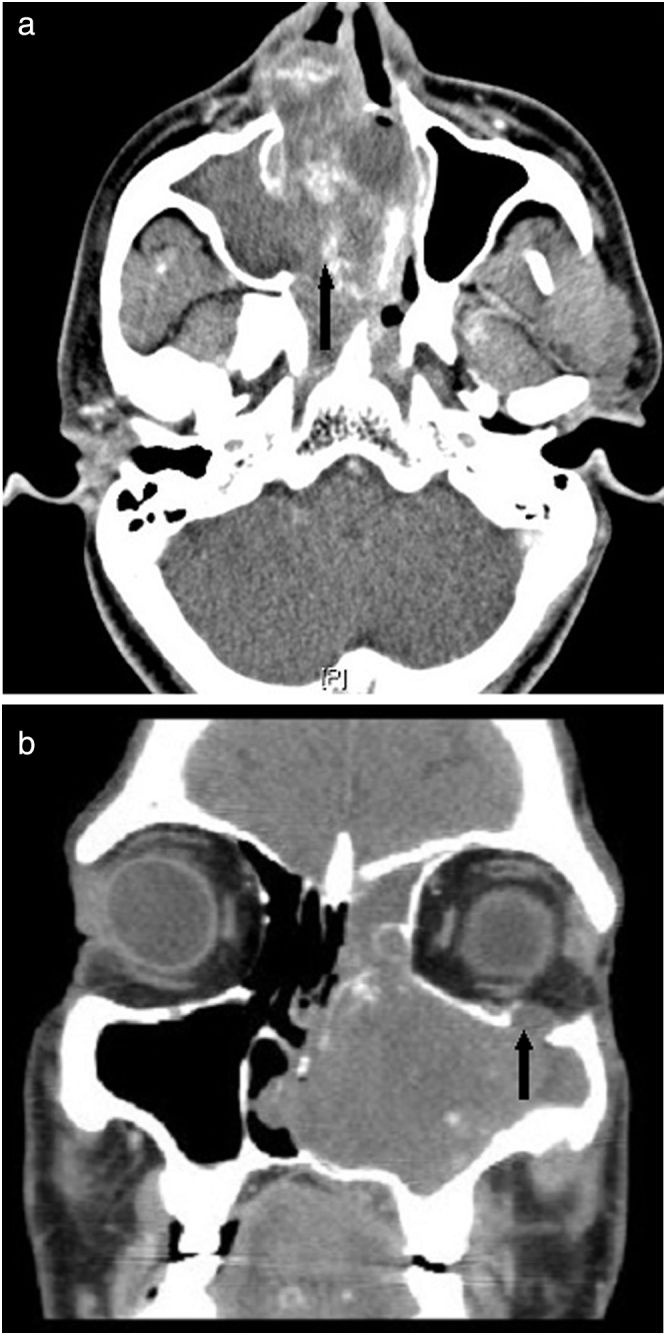
(a) Contrast-enhanced CT scan of the paranasal sinuses axial view showing heterogeneous, soft tissue opacification (black arrow) filling the right maxillary sinus and right nasal cavity pushing the septum to the left. (b) CT scan of the paranasal sinuses coronal view showing expansion of the left maxillary sinus cavity, bone remodelling, widening of the infundibulum and erosion of the orbital floor (black arrow).

Contrast-enhanced CT scan of the paranasal sinuses was obtained for each patient before surgery. Mild to moderately enhancing, heterogeneous, soft tissue opacification filling the sinuses was observed in all the scans (Fig. 1a). In addition, 80% scans showed multiple intralesional areas of calcification. Table 2 depicts the various sites of involvement.

**Table 2:** Radiological profile of the study patients (*n* = 10).

Case n	CT scan		
	sites involved	Bone erosion	Intralesional hyperdensities
1	MS + NC	No	No
2	MS + AE + PE + FS + NC + NPX	Yes (LP + hard palate)	Yes
3	MS + AE + PE + FS + SS + NC	Yes (orbital floor)	Yes
4	MS	No	Yes
5	MS + AE + PE + NC	Yes (LP + orbital floor + hard palate + anterior and posterior wall MS)	Yes
6	MS	No	Yes
7	MS + AE + PE + NC	No	Yes
8	MS	No	No
9	MS	No	Yes
10	MS + NC	No	Yes

NC, Nasal Cavity; MS, Maxillary Sinus; AE, Anterior Ethmoid sinuses; PE, Posterior Ethmoid sinuses; FS, Frontal Sinus; SS, Sphenoid Sinus; LP, Lamina Papyracea; NPX, Nasopharynx.

The maxillary sinus was involved in all cases with a unilateral soft tissue density mass causing expansion of the sinus, cortical thinning, bone remodelling and widening of the infundibulum in all patients (Fig. 1a). In four patients (40%), extension of the mass into the anterior and posterior ethmoid sinuses was noted. The frontal sinus was involved in two patients and the sphenoid sinus in one patient. Bony erosions of the sinus walls were evident in three patients (30%). In one patient (Case 5), there was extensive bony destruction (Fig. 1b). The mass (6.2 × 4.5 × 5.6 cm in size) was seen eroding all walls of the left maxillary sinus, lamina papyracea, orbital floor and hard palate. The lesion was extending into the pterygopalatine and infratemporal fossae eroding the lateral pterygoid plate, superiorly extending into the orbit abutting the inferior rectus and inferior oblique muscles and anteriorly extending into the premaxillary region and subcutaneous plane.

### Treatment and intraoperative findings

All 10 patients underwent complete excision of the lesion under general anaesthesia. Eight patients underwent endoscopic excision alone while two patients underwent excision via a combined endoscopic and sublabial approach (Table 3). Inferior turbinectomy was combined with excision of the mass in four patients to provide adequate airway, as the inferior turbinate was floppy and medialised due to mass effect. In most patients (80%), the sinonasal mass was friable and necrotic and interspersed with blood clots. There was a polypoidal mass in one patient and a cystic mass with blood clots in another patient. The lesion did not involve or infiltrate the sinonasal mucosa in any patient and could be easily separated from it. The sinonasal mucosa appeared mildly oedematous and was sent separately for histopathology.

**Table 3:** Surgical profile and outcomes of the study patients (n = 10).

Case n	Treatment		Follow up	
	Surgery	Intraop findings	Symptoms at 6 months	Endoscopic finding
1	ESS + inferior turbinectomy	Friable mass filling MS	Nil	No disease
2	ESS	Polypoidal mass filling sinuses and nasal cavity	Nil	–
3	ESS + CL + inferior turbinectomy	Friable necrotic mass with blood clots	Nil	–
4	ESS	Friable necrotic mass filling MS	Nil	No disease
5	ESS + CL + inferior turbinectomy	Fleshy necrotic mass	Nil	–
6	ESS	Blood clot, cystic swelling with solid component	Nil	No disease
7	ESS + inferior turbinectomy	Black fleshy friable mass, septal perforation	Nil	No disease
8	ESS	Fleshy mass	Nil	No disease
9	ESS	Fleshy polypoidal gritty mass	Nil	–
10	ESS	Yellowish fleshy necrotic mass	Nil	–

ESS, endoscopic sinus surgery; CL, Caldwell Luc.

### Histopathology

**Figure 2:**
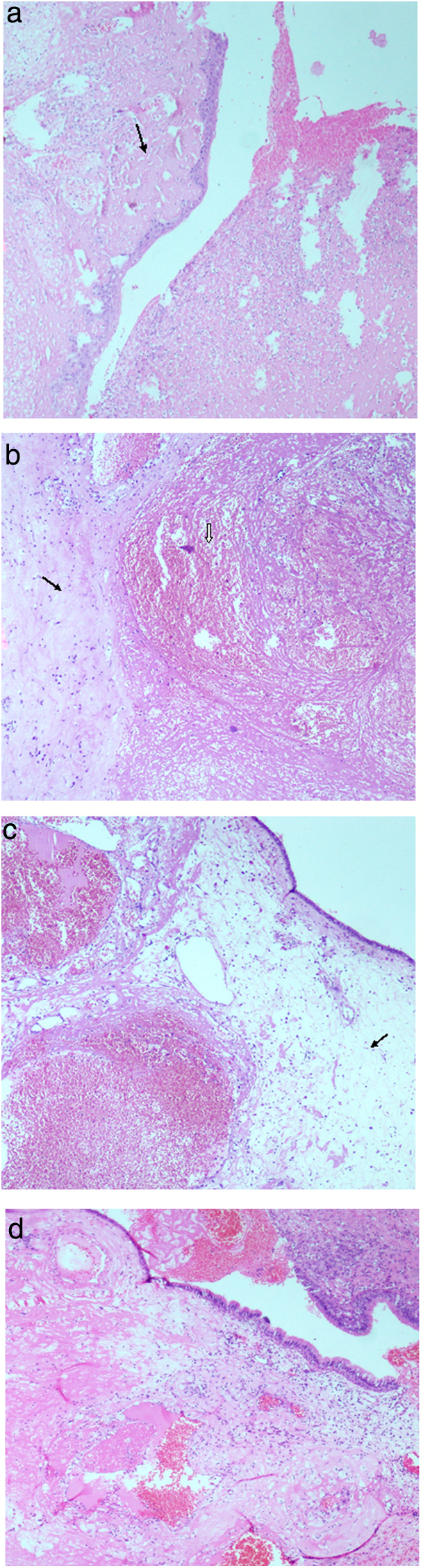
(a) Squamous metaplasia and ulceration replaced by acute inflammatory exudate, arrow pointing towards subepithelial fibrinous exudate (H&E stain at 40×). (b) An ectatic blood vessel with organising thrombus (arrow head) and arrow pointing towards adjacent area of stromal fibrosis (H&E stain at 40×). (c) Respiratory mucosa with marked subepithelial oedema (arrow) and ectatic blood vessels with thrombosis (H&E stain at 40×). (d) Polypoidal respiratory mucosa with subepithelial oedema, marked fibrin exudation and areas of recent haemorrhage (H&E stain at 40×).

Histopathological examination of the specimens showed a polypoidal mass with overlying mucosal ulceration and acute inflammatory exudates. Viable respiratory mucosa usually showed squamous metaplasia in foci (Fig. 2a). The histopathological findings in the subepithelial stroma were a combination of haemorrhage, oedema, infarction, fibrin exudate, stromal hyalinisation and vascular proliferation with ectatic vascular channels and organising thrombi (Fig. 2b–d). Old haemorrhage with haemosiderin deposits and focal dystrophic calcification were seen in a few cases. Many had associated inflammatory granulation tissue with moderate-to-dense mixed inflammatory infiltrates. Occasional multinucleate giant cells and cholesterol clefts were also noted in a case each. Although surface bacterial colonisation was seen, fungal organisms were not demonstrated. There was no cellular atypia. Associated inflammatory polyps and features of mild-to-moderate chronic sinusitis were seen in most of the patients (70%).

### Follow up

The immediate postoperative period was uneventful in all the patients. None of the patients had excessive haemorrhage in the perioperative period or required blood transfusion. All patients were asymptomatic at six months and five patients who underwent postoperative rigid nasal endoscopy had well mucosalised maxillary sinuses with no evidence of residual disease.

## Discussion

OH is a rare, benign condition with locally aggressive behaviour. The pathogenesis of the lesion is haemorrhage into a sinus (typically, the maxillary sinus) and formation of a chronic haematoma. This is followed by organisation through fibrosis and neovascularisation. Song et al.^4^ have described “organization” as “replacement of blood clots by fibrous tissue” and introduced the terminology “organising haematoma of the maxillary sinus”. The previously used terminologies were haematoma,^2^ haematoma-like mass,^1^ pseudotumour^3^ and organised haematoma. The cause for haemorrhage is often unclear. All except two of our patients were hypertensive and this could be a cause for the bleed. Two patients who were hypertensive had coagulation disorders, further increasing their risk for an intrasinus bleed. When bleeding occurs within the nasal cavity, the clots are easily expelled either by forceful blowing of the nose by the patient, manual removal or ciliary action along with mucus. In contrast, when bleeding occurs into a closed sinus, particularly if the blood clot is large, a chronic haematoma results. This haematoma then gets replaced by fibrous tissue and newly formed blood vessels, leading to the formation of OH.

Most reports suggest that the commonest paranasal sinus to be affected is the maxillary sinus.^5^, ^6^ Obstruction of the sinus ostium leads to negative intraluminal pressure and decreased ventilation.^7^ In our study, the maxillary sinus was involved in all the patients. Additionally, in some patients the lesion extended beyond the confines of the maxillary sinus. Previously, only three cases (one involving frontal sinus and two involving sphenoid sinus -) of extra maxillary sinonasal OH have been reported.^5^ We report four new cases of extra-maxillary involvement of OH involving the anterior and posterior ethmoid sinuses (*n* = 4), frontal (*n* = 2) and sphenoid sinuses (*n* = 1). The mean age of presentation in our series was 47.4 years with a male predominance, which is similar to that reported in other studies.^4^, ^5^, ^7^, ^8^

The aetiopathogenesis of OH is still not clearly understood. Accumulation of blood in the maxillary sinus is believed to be the trigger for OH. The cause of bleeding into the sinus may be trauma, surgery, bleeding diatheses or a haemorrhagic lesion within the sinus. Some authors have suggested that either a ruptured aneurysm of a medium-sized vessel related to the affected sinus or inflammatory erosion of an arterial wall may be causative as well.^8^ The aetiological factors related to this could be aggressive fungal infection, radiation therapy and recurrent epistaxis.^7^, ^9^ In a study by Choi et al.,^5^ about 30% (6 out of 17 patients) of patients with OH were hypertensive and were on aspirin. The antiplatelet agent was proposed as a possible causative factor in these patients. In our study, 80% of the patients were hypertensive. Among those who had hypertension, one patient had factor XI deficiency and another. had thrombocytopenia. The effect of these comorbidities could have been cumulative. None of the patients in our series were on any antiplatelet medications. Three patients, however, gave history of recent nasal surgery and this could also have been a cause for haematoma formation. In view of the high prevalence of hypertension among our series of patients diagnosed with OH, we believe that hypertension itself may be a risk factor for developing OH.

A number of theories have been proposed to explain pathogenesis of this condition.^7^, ^8^, ^9^, ^10^ The “negative spiral theory” proposed by Omura et al.^10^ is based on immunohistopathological evidence,^11^ and is the most accepted theory at present. Collection of blood in the paranasal sinuses with poor sinus ventilation and drainage can lead to haematoma formation, which remains in the sinus for a long time. As part of the biological healing processes, necrosis, fibrosis and hyalinisation occur leading to a capsule formation around the haematoma, thus preventing its reabsorption. Later, neovascularisation develops within the capsule, where the new vessels are weak, and rebleeding might easily occur. Recurrent intracapsular bleeding, leads to the eventual formation of OH. Progressive expansion causes pressure demineralisation of adjacent bony sinus walls, leading to remodelling and subsequent bony erosion. Imayoshi et al.^12^ have observed that vascular endothelial growth factor (VEGF) and its receptors (VEGFR2) are related to the neovascularisation seen in OH.

The radiological appearance of sinonasal OH is rather nonspecific. On CT scans without contrast the lesion is seen as a large mass causing expansion of the maxillary sinus with bony erosion and various degrees of heterogeneous high attenuation within the lesion. Post contrast, patchy heterogeneous enhancement is seen, probably due to the neovascularisation.^7^ The surrounding inflammed sinus mucosa in spite of the bony erosions points towards a benign process.^9^ On MRI scanning, the lesion is heterogeneous in signal intensity on both T1 and T2 weighted images and is always well demarcated from the surrounding structures. The heterogeneous signal intensity reflects the various components contained within the lesion, such as haemorrhage in various stages, fibrosis, and varying amounts of vascular proliferation. T2 weighted images demonstrate a hypointense peripheral rim which corresponds histologically with an attenuated fibrous pseudocapsule. This biphasic appearance is an important imaging characteristic of OH.^4^ Hur et al.^13^ have demonstrated irregular nodular, frond-like, papillary or cerebriform enhancement in all their cases.

OHs are diagnostic dilemmas clinically and radiographically, mimicking benign or malignant neoplastic processes. Various differential diagnoses to be considered for unilateral mass in the sinonasal cavity detected on CT and MR images include mucocoele, fungus ball, inflammatory polyp, cholesterol granuloma, inverted papilloma, haemangioma, and carcinoma. Contrast-enhanced CT or MRI scanning of the paranasal sinuses is extremely useful in excluding mucocoele, fungal ball, inflammatory polyp, and cholesterol granuloma as they do not usually enhance. Inverted papilloma primarily shows a characteristic convoluted cerebriform pattern on T2- or enhanced T1-weighted MR images. Clear cut bony destruction associated with adjacent tissue invasion is a hallmark of carcinoma. In contrast, OH typically shows thinning, expansion and smooth erosion of the sinus walls. The most difficult lesion to differentiate from OH both clinically and radiologically is sinonasal haemangioma, especially the cavernous type.

On histopathological examination OH cases show a combination of vascular ectasia, recent and old haemorrhage, oedema, fibrin exudation, fibrosis and hyalinisation and neovascularisation. Occasional cases may show some inflammatory granulation tissue in the subepithelial tissue. The primary histopathological differential diagnosis considered in the present series was haemangioma. Although dilated blood vessels and vascular proliferation which occur in haemangioma were seen in the cases studied, the presence of abundant fibrin deposits with hyalinisation, haemorrhage and neovascularisation precluded the diagnosis. Surgical samples were processed in entirety to avoid missing any overt focus of atypia or malignancy. Fungal stains were done to exclude any invasive fungal sinusitis. Despite the presence of clinical and radiological features which bear a strong resemblance to malignancy, a negative histopathology result does not warrant further surgery. A high index of suspicion with careful histopathological examination is essential to arrive at the diagnosis.

The treatment of OH is complete surgical excision. Various approaches such as lateral rhinotomy, Caldwell-Luc, Denker's surgery, combined endoscopic and Caldwell-Luc approach and endoscopic sinus surgery have been described.^1^, ^4^, ^5^ Only 2 of our patients required a combined approach (Caldwell Luc plus endoscopic sinus surgery) to completely remove the disease. Most patients (80%) were managed by an endonasal endoscopic approach alone. We noted that we adopted a less invasive procedure with each subsequent case in our series and this could be attributed to increased awareness of the condition with time.

## Conclusion

Sinonasal OH is a rare, benign, locally aggressive disease which mimics sinonasal malignancy both clinically and radiologically. Histopathology is confirmatory. Complete endonasal endoscopic surgical excision is sufficient in the majority of patients.

## Ethical approval

Since this is a retrospective study, informed consent was not required. However, the institutional review board approval was obtained.

## Conflicts of interest

The authors declare no conflicts of interest.
